# Evaluating and modeling of photon beam attenuation by a standard treatment couch

**DOI:** 10.1120/jacmp.v12i4.3561

**Published:** 2011-07-12

**Authors:** Zhihui Hu, Jianrong Dai, Liang Li, Yin Cao, Yixin Song, Guishan Fu

**Affiliations:** ^1^ Department of Radiation Oncology Cancer Institute & Hospital, Chinese Academy of Medical Sciences Beijing China

**Keywords:** treatment couch, beam intersection, beam attenuation, model‐based segmentation, two‐dimensional ionization chamber array

## Abstract

The purpose of this study was to evaluate beam attenuation by treatment couch and build a treatment couch model in TPS to check for beam–couch intersection at the planning stage and deal with beam attenuation by treatment couch in dose calculation. In this study, a standard treatment couch, Siemens ZXT couch, has been incorporated into ${\text{Pinnacle}}^{3}$ 8.0 TPS, based on an existing TPS tool, model‐based segmentation (MBS). This was done by generating the couch's model from contours of the couch, together with the density information. Both the geometric and dosimetric accuracy of the couch model were evaluated. The test of beam–couch intersection prediction showed good agreement between predicted and measured results, and the differences were within 1° gantry rotation. For individual posterior oblique beams, the attenuation by metallic frames and PMMA couch top could reach nearly as high as 60% and 10%, respectively. For several posterior oblique beams (180°, 220°, 235°) that attenuated by the PMMA couch top, the calculated and measured dose distributions were compared. The dose differences at central axis were within 1%, and almost all points agreed with the calculations when the DD and DTA criteria of 3%/3 mm were adopted. The difference between calculated and measured attenuation factors were within 0.5%. This study demonstrates that the couch model created by MBS, which contains geometric and density information of the couch, can be used to detect the beam–couch intersection, and also is able to provide an accurate representation of the couch top attenuation properties in patient dose calculation.

PACS numbers: 87.55.D‐, 87.55.Gh, 87.55.km

## I. INTRODUCTION

Combinations of several beams from different directions are commonly used in external beam radiation therapy to produce a highly conformal dose distribution, but also with an increased possibility that the radiation beams intersect with the treatment couch.^(^ ^1^ ^–^ ^3^ ^)^ A number of investigations about the attenuation properties of different treatment couches have been reported.^(^ ^4^ ^–^ ^9^ ^)^ The attenuation by carbon fiber couches was found to be less than 3% at normal incidence for 6 MV photon beams, depending on the couch design.^(^ ^7^ ^–^ ^9^ ^)^ McCormack et al.^(^ ^9^ ^)^ demonstrated the effect of gantry angle on attenuation by a carbon fiber couch, ranging from 2% at normal incidence to 9% at 70° incidence for 6 MV photon beams. Prior to the use of the carbon fiber, the conventionally used materials for treatment couch top were PMMA and wood, which cause more attenuation than carbon fiber. De Ost et al.^(^ ^10^ ^)^ showed the attenuation up to 4% by either 12.5 mm PMMA plate or 19 mm wooden hardboard at normal incidence for 6 MV beams. Munjal et al.^(^ ^11^ ^)^ observed the attenuation by a 12 mm PMMA plate ranging from 4.85% at normal incidence to 10.55% at 60° incidence.

However, most of the commercial treatment planning systems (TPSs) don't adequately take account of beam attenuation by treatment couch. Thus, if the posterior oblique beams pass through the treatment couch before arriving at the target, the dose delivered to the patient would be lower than the planned one. The situation could be worse for the standard treatment couches which use the metallic frame and spine for strength. To avoid a large attenuation by those metallic components, the radiation therapists have to check for a possible intersection between the beams and the metallic components prior to patient pretreatment setup. To save time on the treatment unit, the check should be done at the treatment planning stage. Therefore, it is desirable to build a treatment couch model in TPS to check for beam–couch intersection during the planning process and deal with couch attenuation in dose calculation.

Recently, different approaches were developed for incorporation of the treatment couch into a TPS. Spezi et al.^(^ ^12^ ^)^ edited each slice of the patient CT with an in‐house software to replace the CT couch with the treatment couch. Mihaylov et al.^(^ ^13^ ^)^ used contours with appropriate densities to simulate the treatment couch geometry in Pinnacle TPS, realizing through scripting language. Van Prooijen et al.^(^ ^14^ ^)^ transferred the couch's contours to patient CT based on a fusion tool. These investigations showed that the difference between calculated and measured dose could be reduced to within 2% after incorporating the treatment couch into the TPS.

In this study, we investigated the attenuation properties of a standard treatment couch, Siemens ZXT system, together with the PMMA couch top which is supposed to be less radio‐transparent than carbon fiber couch but is still in use for radiation therapy in many clinics. We also developed a simple method of incorporating the treatment couch into treatment planning. Our method is based on an existing TPS tool, model‐based segmentation (MBS),^(^ ^15^ ^–^ ^18^ ^)^ an automatic organ delineation tool. The MBS tool uses a triangular surface mesh to model an organ's shape. The organ's mesh is stored in TPS model library and can be loaded at any stage of the planning process. Therefore, the MBS tool can be used for incorporating the treatment couch into treatment planning.

## II. MATERIALS AND METHODS

### A. Treatment couch structure

**Figure 1:**
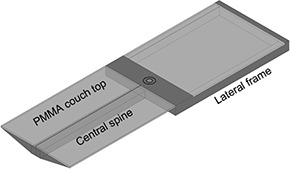
Schematic image of the ZXT system.

The ZXT treatment couch (Siemens AG, Erlangen, Germany) was investigated in this work. It is part of the Siemens Primus radiation therapy system. As shown schematically in Fig. 1, the frame of the ZXT couch consists of two sections, the central spine and the lateral frame (denoted hereafter as ‘section A’ and ‘section B’), which both are metallic. Either section can be used, depending on the beam arrangement. The detachable PMMA plate couch tops are mounted on the ZXT system to support the patient. The couch top mounted on section A is constructed by two PMMA plates (each is 24.5 cm wide), with a gap of 0.5 cm in the lateral direction, whereas the PMMA plate mounted on section B is 49.5 cm wide. The thickness of these PMMA plates is 1.3 cm, and their density, derived from the CT image set, is $1.10\;g/{\text{cm}}^{3}$.

### B. Modeling of treatment couch in TPS

**Figure 2:**
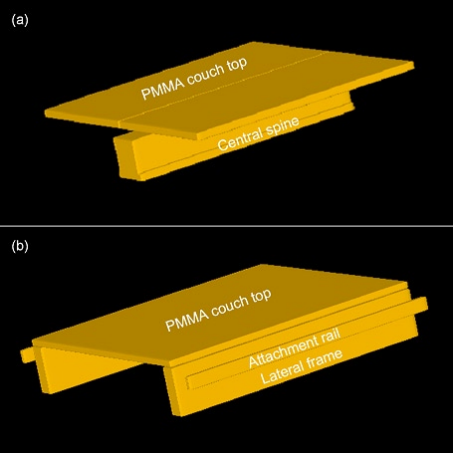
Surface meshes for ZXT treatment couch models: section A (a) and section B (b).

The ZXT couch was scanned by Philips Big Bore Brilliance CT with slice thickness of 5 mm, and then the CT dataset was transferred to ${\text{Pinnacle}}^{3}$ 8.0 TPS (Philips Medical Systems, Fitchburg, WI). For each ZXT section, the outlines of the couch that include PMMA plates and metallic frames were contoured manually at the central slice of the section and then copied to other slices of this section. The shape and dimensions of the couch were determined by the data in vendor‐supplied user manuals, and the results measured with a ruler. For each section, a surface mesh was generated from the contours by MBS tool. Then, the meshes of the two couch sections, together with the density information, were stored into the MBS model library in ${\text{Pinnacle}}^{3}$. Figure 2 shows the TPS 3D visualization of surface meshes for section A and section B couch models.

**Figure 3:**
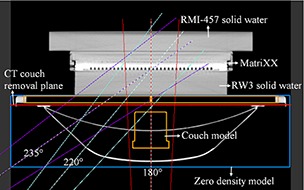
Screen capture from the TPS showing the incorporation of treatment couch into TPS and setup of IMRT MatriXX for measurements and calculations. The MatriXX is placed in prone position over the RW3 solid water slabs. A solid water slab (RMI‐457) is placed above the back face of the MatriXX for immobilization and backscatter.

Figure 3 shows the incorporation of the couch model into a treatment plan. First, a mesh with cuboid shape and zero density (denoted as ‘zero density model’) is loaded from the MBS library and placed under the phantom/patient to remove the CT couch. Then, we loaded the treatment couch model and placed it at the location where the CT couch is and centered laterally on CT slice. Thus, the treatment couch model is overlapped with the zero density model, and the overlapped area is overwritten with the density of PMMA couch top.^(^ ^13^ ^)^ Note that, in Pinnacle, the area below the CT couch removal plane (Fig. 3) will be treated as air. In this study this removal plane was placed below the PMMA couch top but above the ZXT metallic frames in treatment planning, so the attenuation property of the PMMA couch top was considered in dose calculation, while the attenuation by the metallic frames was not.

### C. Test of beam–couch intersection prediction

In this study, measurements and TPS calculations for 10 setups with different vertical and lateral couch displacements were performed both on section A and B, to test the accuracy of the prediction of beam intersection with metallic frames. The beams would probably intersect with the central spine for tests on section A, whereas intersect with the lateral frames for tests on section B. One edge of a $10\times 10\;{\text{cm}}^{2}$ light field was used for visual check. A cross‐comparison, between calculated and measured gantry angles that any part of the beam is just not clipped by the couch's metallic frames, were accomplished.

### D. Validation of dose calculation with couch model

Dose verification was performed with a 2D array of ion chambers, MatriXX (IBA Dosimetry GmbH, Germany). MatriXX consists of 1020 vented ionization chambers, arranged in a $24\times 24\;{\text{cm}}^{2}$ matrix, with a distance of 0.762 cm between any two neighboring chambers.

Measurements for three posterior oblique fields (180°, 220°, 235°) that passed through treatment couch top were performed, both on section A and B. As shown in Fig. 3, the MatriXX was placed in a prone position and irradiated at 100 cm source‐to‐detector distance with 5 cm solid water (RW3) as buildup material. A $10\times 10\;{\text{cm}}^{2}$ 6 MV beam with 100 MU exposures was used.

Measurements of dose distributions without attenuation by treatment couch at different beam angles of incidence were also performed when the MatriXX was in supine position and the beam angles were set to 0°, 40°, and 55°, respectively. By computing the percentage difference between measurements made with and without couch attenuation, the attenuation images for treatment couch components were derived.

The MatriXX, together with the RW3 solid water slabs, was CT‐scanned under identical geometry setup of measurements, and then imported into the TPS. Treatment plans with couch models were designed. Since the metallic supporting frames of treatment couch can cause serious beam attenuation, it is meaningless to take account of their attenuation in dose calculation. A better way is to avoid the beam intersection with these metallic components at the planning stage. Therefore, only the attenuation by PMMA couch top was considered in TPS calculations. A cross‐comparison between calculated and measured dose distributions was performed using OmniPro IMRT software (version 1.6, IBA Dosimetry GmbH, Germany) in the absolute dose comparison mode. The calculated dose distributions had a point spacing of $1\times 1\;{\text{mm}}^{2}$. No interpolation was applied to the dose distributions measured with the MatriXX. A gamma index histogram was used for quantitative analysis of the 2D absolute dose distributions.^(^ ^19^ ^–^ ^21^ ^)^ Commonly‐used percent dose difference (DD) and dose to agreement (DTA) criteria of 3%/3 mm and 2%/2 mm were adopted for comparison. Dose difference at central axis (CAX) was calculated with respect to the measured dose.

The TPS‐calculated attenuation factors for PMMA couch top at different beam incidence were also derived. Comparisons between measured and calculated attenuation factors were completed.

## III. RESULTS

### A. Test of beam–couch intersection prediction

The test of beam–couch intersection prediction showed good agreement between predicted and measured results. Both for beam intersection with lateral frame and central spine, the differences of the gantry angle were within ±1°, which could be considered acceptable for intersection prediction.

### B. Validation of dose calculation with couch model

**Figure 4:**
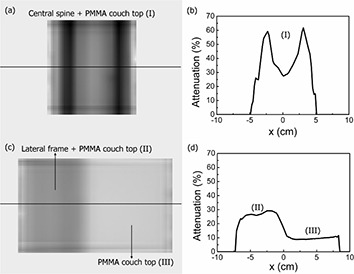
Attenuation images and attenuation profiles along the x‐axis for metallic frames and PMMA couch top: (a) and (b) for central spine at normal incidence, (c) and (d) for lateral frame and PMMA couch top at 55° incidence.

Figures 4(a) and (b) show the attenuation image and attenuation profile for central spine, together with PMMA couch top at normal incidence. The attenuation by central spine is not uniform because of the spine's hollow structure. It is about 30% at the central area and reaches as high as 62% when the beam is attenuated by the spine's edge. Figure 4(c) shows the attenuation image for lateral frame at 55° incidence. The darker areas in the image indicate the region where the beam is attenuated by the lateral frame. The lateral frame, together with the PMMA couch top, attenuates the beam by about 27%. As shown in Fig. 4(d), the attenuation by PMMA couch top varies between the center and inner periphery of the beam, and it is dependent on the distance from the central axis. This effect is due partly to the beam‐hardening effect of the flattening filter, and partly to the fact that the radiation path length through PMMA also varies between the center and inner periphery of the beam.

Table 1 presents the comparison between calculated and measured attenuation factors for three posterior oblique fields that passed though the PMMA couch top but not intersected with the metallic supporting frames. Both the measured and calculated attenuation factors were computed over the central 80% of the field size in order to exclude the penumbral effects. The measured attenuation factor at central axis (CAX) for normal incidence is consistent with the published results.^(^ ^10^ ^,^ ^11^ ^)^ As would be expected, attenuation increases with increasing beam angle of incidence and reaches as high as 8.7% at CAX at 55° incidence. Table 1 also shows good agreement between the measured and calculated attenuation factors at CAX, as well as the mean attenuation over the central 80% of the field size. The largest difference of attenuation factor at CAX, as well as the mean attenuation, is within 0.5%. This accuracy is consistent with the results achieved with a different method to incorporate the treatment couch into ${\text{Pinnacle}}^{3}$ TPS.^(^ ^12^ ^,^ ^13^ ^)^

**Table 1:** Comparison between calculated and measured attenuation factors for three posterior oblique fields. CAX represents central axis of the beam.

*Gantry Angle*	*Measured Attenuation*			*Calculated Attenuation*		
	*CAX (%)*	*Mean (%)*	*Range (%)*	*CAX (%)*	*Mean (%)*	*Range (%)*
180°	4.2	4.5	3.9~5.5	4.3	4.7	4.2~5.6
220°	5.8	6.2	5.6~7.3	6.2	6.7	6.1~7.8
235°	8.7	9.7	8.5~12.3	8.7	9.6	8.5~12.4

Table 2 shows the comparison of calculated and measured dose distributions for the above three posterior oblique fields that passed though the PMMA couch top. For the routine clinical situation where the couch top attenuation was ignored, the PMMA couch top introduced a dose difference up to 8.8% at CAX, thus leading to the low passing rates for the plans calculated without couch attenuation. When the couch top was included into dose calculations, the dose difference at CAX was improved to within 1%, which is comparable to the TPS dose calculation accuracy of an open field.^(^ ^22^ ^)^ Almost all points agreed with the calculations when the DD and DTA criteria of 3%/3 mm were adopted. Even with the more stringent criteria of 2%/2 mm, good passing rates were obtained for all the comparisons. These results demonstrate good accuracy of the TPS when including the couch top into dose calculation.

**Table 2:** Comparison of measured dose distributions with calculated planar doses which were computed with (WI) or without (WO) couch top attenuation for different posterior fields.

			*Gantry Angle*			
	*180°*		*220°*		*235°*	
	*WI*	*WO*	*WI*	*WO*	*WI*	*WO*
Dose Difference (CAX)	0.7%	3.6%	0.9%	5.4%	0.7%	8.8%
2%DD 2 mmDTA	98.0%	34.9%	97.6%	26.1%	98.2%	26.7%
3%DD 3 mmDTA	99.8%	56.5%	100%	49.5%	100%	41.5%

## IV. DISCUSSION

To our knowledge, the best method of incorporating the treatment couch into dose calculation is using identical couch top both for patient CT scan and treatment, but it is unrealistic in our institute. A “simple” solution reported by McCormack et al.^(^ ^9^ ^)^ calls for a fixed posterior oblique beam, using a correction factor based on the CAX attenuation to adjust the beam's MU. However, this process may not accurately predict the treatment couch's attenuation properties. First, for beams passing through the edge of the couch top and partially attenuated by the couch top, adjusting the MU based on CAX attenuation may result in a dose increase to the volume irradiated by the unattenuated portion of the field. Second, as mentioned above, the attenuation factor varies between the center and inner periphery of the beam. This is more pronounced for oblique beams with a large angle of incidence. As shown in Table 1, at gantry angle of 235°, the measured attenuation factor at CAX is about 3.6% lower than the largest attenuation over the central 80% of the field size. Therefore, simply adjusting the beam's MU based on the attenuation factor at CAX may result in an underestimated dose distribution at the inner periphery of the beam. In this investigation, we developed a method to achieve the realistic simulation of the treatment couch in ${\text{Pinnacle}}^{3}$ TPS, and we have shown its effectiveness in predicting the beam intersection with metallic frames and correcting the couch top attenuation. The process of creating couch model, as well as importing the couch model for treatment planning, is simple. The importing process requires minimal additional time at the planning stage.

In some treatment plans, some posterior oblique beams are likely to intersect with the metallic supporting frames. Without the couch model, these plans require an extra procedure to check for these potential intersections prior to patient pretreatment setup, and it certainly increase the load on treatment unit. If the intersections indeed exist, replanning would be needed. Therefore, we expect to use the couch models to avoid the beam–couch intersection at the planning stage. First, the models are used to determine whether the treatment can take place on central spine section or lateral frame section without the beam intersection with metallic frames. Second, if intersections exist both in central spine and lateral frame cases, the adjustment of the initial beam arrangement would be required. In such a way, it may save considerable time on treatment unit. Due to the patient setup error on treatment unit, the indexed immobilization devices are required to reduce the lateral displacement in patient setup. If an immobilization device is indexed to the couch top, it will be fixed with respect to the couch top for each fraction. Previous studies showed that with the indexed immobilization devices, the patient setup error could be within 0.3 cm.^(^ ^23^ ^–^ ^24^ ^)^ Furthermore, an appropriate safety margin between the radiation field edge and metallic frames should be considered when defining the beam arrangement in treatment planning.

If treatment takes place on center spine side, for beams that partially attenuated by the edge of the PMMA couch top, the lateral displacement of the patient may result in a dose discrepancy between delivery and prescription. For plans that have multiple beams, the discrepancy may be negligible. However, for plans that have only a very small number of beams, the discrepancy may be more pronounced, depending on the beam weighting. Therefore, for single‐beam plans or plans having a very small number of beams, a possible solution is to avoid such partial attenuation at the planning stage. This can be achieved in a way similar to that of checking for beam–couch intersections.

Using the couch models in dose calculation also requires that the field of view (FOV) of the patient/phantom CT is larger than the width of the couch top. Otherwise, for the parts of the couch top which are extending beyond the edges of the CT slices, their density information will not be taken into consideration by Pinnacle; thus, the beam attenuation by those parts will also not be taken into consideration in dose calculation. It may make some incidences unacceptable. Nevertheless, in ${\text{Pinnacle}}^{3}$, the visualization of the MBS model of the treatment couch is not limited by FOV. Therefore, if the couch model is only used for checking the beam–couch intersection, the size of FOV is not important.

## V. CONCLUSIONS

This study has investigated the attenuation properties of a standard treatment couch and developed a simple method to incorporate the couch into TPS. Beam attenuation through the metallic supporting frames was significantly large; thus the intersection between posterior oblique beams and these highly attenuating couch components should be avoided. MatriXX measurements showed the attenuation by PMMA couch top could reach nearly as high as 10% for individual oblique beams. If the attenuation by PMMA couch top was included in TPS dose calculation, the calculated dose distributions agreed well with the measured data, and the treatment dose error could be reduced to less than 1% at CAX. However, clinical implementation requires the indexed immobilization devices to achieve the accuracy of modeling the treatment couch in TPS.

## ACKNOWLEDGMENTS

The authors would like to acknowledge the IBA Dosimetry for providing a MatriXX array for this work.
